# We need to talk about shrooms

**DOI:** 10.1177/14550725251341722

**Published:** 2025-05-21

**Authors:** Matilda Hellman

**Affiliations:** Uppsala University, Uppsala, Sweden; 3835 University of Helsinki, Helsinki, Finland

**Keywords:** psychedelics, hippies, psilocybin, critical research, hallucinogens

For the hippie movement, psychedelics were a symbol of peace, love and consciousness expansion, carrying the potential to liberate humanity from destructive norm-regulated and moralistic constraints. A significant part of the public ‘othering’ of the rebellious youth movement of the 1960s revolved around the threat to social order resulting from the use of hallucinogens and psychedelic drugs. The typical account of the 1960s psychedelics era ends with a culmination in the Manson murders and the targeted strict regulations and adherent moral panic of the drug prohibition of the 1970s and 1980s. (Weaver, 2024)

Once symbols of countercultural rebellion and deviance, substances such as psilocybin have now re-entered the mainstream – this time escorted by peer-reviewed studies, white papers, TED Talks and a massive interest from the pharma industry (Aday et al., 2023). Psilocybin in particular is emerging as a flagship of what has been dubbed the psychedelic renaissance. This cultural and scientific rehabilitation of psychedelics is indeed among the most remarkable epistemic shifts in contemporary mental health discourse. The cultural rebranding of psychedelics – “the psychedelic turn” – is a reconfiguration of what counts as healing, knowledge and consciousness. Substances once seen as threats to public order are now being framed as novel and precise psychiatric tools – potential cures for depression, trauma, addiction and even existential distress. (Kugel et al., 2025). Yet. as this new era unfolds, the role of social scientists is naturally to be critical in all directions: what is being remembered and what is being forgotten? How is the evidence built and used for different reasons?

The social imaginary of psychedelics has tended to oscillate between utopia and dystopia. This is indeed a fruitful point of departure for critical drug researchers, who are provided room to investigate how societies take part in, react upon and adapt to different materializations of drugs and drug use. Today, we are witnessing a great medicalization in tandem with mental health awareness and increasing repertoire of addictive behaviours in the prosperous world. The role of psychedelics in natural remedies and indigenous cultures is likely to become less emphasized as big capital and technology start to dominate its significations and cultural position.

The psychedelic revival is underpinned by clinical self-technological innovation combined with a celebrity and mass-media induced psy-care logic. Research institutions, private clinics and biotech startups are driving this transformation, promising a new frontier of psychedelic-assisted therapy that could, in the words of some advocates, revolutionize mental health care (Jelmini, 2025)

From a social science perspective, however, one-sided optimism should signal caution and careful scrutiny. While some clinical studies have reported impressive outcomes – particularly in treatment-resistant cases, there is a growing risk that the current wave of enthusiasm may re-inscribe a different kind of reductionism. As the neuroscientific framing of psychedelics gains traction, there is a tendency to abstract their effects from context, meaning, and culture and the very social, relational and symbolic dimensions of altered states that the psychedelics use is motivated by in the first place (Kähönen, 2023). Furthermore, the question of how a trip actually works is often answered by invoking neuroplasticity, receptor activity, or brain imaging results – evidence known to contain its own flaws (Rose & Abi-Rached, 2014).

Recent qualitative studies (Bremler et al., 2023) and troubling case reports (Cardone et al., 2025; Müller et al., 2025) remind us that psychedelic experiences are highly variable, can be unpredictable and are highly shaped by personal factors. Pre-existing mental health vulnerabilities, unsafe environments, inadequate therapeutic support and unclear dosing are all “normal” circumstances to take into account in all types of substance consumption, also of those with far less potency and no long-lasting psychiatric consequences. Research with economic ties to the psychedelic pharma industry has debunked the negative publicity as misinformed and anecdotical (Schlag et al., 2022).

This is by no means a call to dismiss psychedelics or psychedelics research, nor to ignore the therapeutic promise of psychedelic use. On the contrary, the transformative potential of psychedelics lies in their resistance to disciplinary boundaries and, in order to harness that potential responsibly, we must resist the temptation to medicalize without context, to promise without caution and to narrate without complexity. If we are to engage with this question ethically and critically, we need to talk – openly, rigorously and reflexively – about shrooms.

## In this issue

In a debate article, Selbekk et al. (2025) discuss gender dynamics in drug-using contexts and its implications for treatment and support. Chrysoulakis et al. (2025) take a look at open drug scenes across cities and Jääskeläinen and Kuusisto (2025) have asked social workers about gambling problems in Tampere, Finland. Trolldal et al. (2025) have studied how alcohol consumption may have been associated with financial and mental health problems in Sweden during the COVID-19 pandemic. Moe et al. (2025) have studied drug-free friendships and substance use among a cohort of individuals with multiple substance use disorders.

## References

[bibr1-14550725251341722] Aday J. S. Barnett B. S. Grossman D. Murnane K. S. Nichols C. D. Hendricks P. S. (2023). Psychedelic commercialization: A wide-spanning overview of the emerging psychedelic industry. Psychedelic Medicine, 1(3), 150–165. 10.1089/psymed.2023.0013 40046566 PMC11661494

[bibr2-14550725251341722] Bremler R. Katati N. Shergill P. Erritzoe D. Carhart-Harris R. L. (2023). Case analysis of long-term negative psychological responses to psychedelics. Scientific Reports, 13(1), 15998. 10.1038/s41598-023-41145-x 37749109 PMC10519946

[bibr3-14550725251341722] Cardone P. Núñez P. Alnagger N. L. Martial C. van der Lande G. J. Sandell R. Carhart-Harris R. Gosseries O. (2025). Psilocybin for disorders of consciousness: A case-report study. Clinical Neurophysiology, 173(3), 181–189. 10.1016/j.clinph.2025.02.264 40147181

[bibr4-14550725251341722] Chrysoulakis A. , et al. (2025). Open drug scenes across city sizes: Socioeconomic status, crime patterns and community perspectives. Nordic Studies on Alcohol and Drugs, 42(3), 210–225. https://journals.sagepub.com/doi/full/10.1177/14550725251327516 10.1177/14550725251327516 PMC11915228 40110533

[bibr5-14550725251341722] Jääskeläinen P. Kuusisto K. (2025). Problematic gambling in municipal social work in Tampere, Finland: Social workers’ perceptions of service pathways before the casino opening and the health and social services reform*.* Nordic Studies on Alcohol and Drugs, 42(3), 226–242. https://journals.sagepub.com/doi/full/10.1177/14550725251325032 10.1177/14550725251325032 PMC11924049 40124887

[bibr6-14550725251341722] Jelmini M. (17 February 2025). Kan partydroger läka en deprimerad hjärna? [Can party drugs heal a depressed brain?] *Svenska Dagbladet*. https://www.svd.se/a/nyMMmL/ecstasy-svamp-ketamin-och-lsd-pa-vag-revolutionera-psykiatrin

[bibr7-14550725251341722] Kähönen J. (2023). Psychedelic unselfing: Self-transcendence and change of values in psychedelic experiences. Frontiers in Psychology, 14(6), 1104627. 10.3389/fpsyg.2023.1104627 37388660 PMC10300451

[bibr8-14550725251341722] Kugel J. Laukkonen R. E. Yaden D. B. Yücel M. Liknaitzky P. (2025). Insights on psychedelics: A systematic review of therapeutic effects. Neuroscience & Biobehavioral Reviews, 173(6),106117. 10.1016/j.neubiorev.2025.106117 40127876

[bibr9-14550725251341722] Moe F. , et al. (2025). Changes in the trajectories of drug-free friendships and substance use among a cohort of individuals with multiple substance use disorders. Nordic Studies on Alcohol and Drugs, 42(3), 257–273. https://journals.sagepub.com/doi/10.1177/14550725251332929 10.1177/14550725251332929 PMC12003346 40255476

[bibr10-14550725251341722] Müller F. Sauer T. Hänny C. Mühlhauser M. Lang U. E. (2025). Suicide of a patient shortly after psilocybin-assisted psychedelic therapy. A case report. Psychiatry Research, 345(3)116381. 10.1016/j.psychres.2025.116381 39889565

[bibr11-14550725251341722] Rose N. Abi-Rached J. (2014). Governing through the brain: Neuropolitics, neuroscience and subjectivity. The Cambridge Journal of Anthropology, 32(1), 3–23. 10.3167/ca.2014.320102

[bibr12-14550725251341722] Schlag A. K. Aday J. Salam I. Neill J. C. Nutt D. J. (2022). Adverse effects of psychedelics: From anecdotes and misinformation to systematic science. Journal of Psychopharmacology, 36(3), 258–272. 10.1177/02698811211069100 35107059 PMC8905125

[bibr13-14550725251341722] Selbekk A. , et al. (2025). Gender dynamics in drug-using contexts: Implication for treatment and support. Nordic Studies on Alcohol and Drugs, 42(3), 204–209. https://journals.sagepub.com/doi/10.1177/14550725251328217 10.1177/14550725251328217 PMC11948265 40160233

[bibr14-14550725251341722] Trolldal B. , et al. (2025). Changes in self-reported alcohol consumption in relation to financial and mental health problems: Experiences from the COVID-19 pandemic in Sweden. Nordic Studies on Alcohol and Drugs, 42(3), 243–256. https://journals.sagepub.com/doi/full/10.1177/14550725251328161 10.1177/14550725251328161 PMC11948224 40160232

[bibr15-14550725251341722] Weaver E. (2024). The Beat Generation’s Influence on the Hippie Movement and Psychedelic Rock. Senior Honors Projects, 2020-current, 169. https://commons.lib.jmu.edu/honors202029/169

